# Spatio-temporal dynamics of ingroup interactions in macaques

**DOI:** 10.1038/s41598-025-16391-w

**Published:** 2025-08-22

**Authors:** Tadeusz W. Kononowicz, Felipe Rolando, Lucas Maigre, Angela Sirigu, Jean-René Duhamel, Sébastien Ballesta, Sylvia Wirth

**Affiliations:** 1 Institut des Neurosciences Paris-Saclay (NeuroPSI), Université Paris-Saclay, CNRS, Saclay, 91400 France; 2 Institut des Sciences Cognitives, UMR5229 CNRS, 67 Boulevard Pinel, Bron, 69500 France; 3 Institut de Neuroscience de la Timone, UMR7289 CNRS, Faculté de Médecine, 27 Bd Jean Moulin, Marseille, 13005 France; 4 Laboratoire de Neurosciences Cognitives et Adaptatives, UMR 7364 CNRS, Strasbourg, France; 5 Centre de Primatologie de l’Université de Strasbourg, Niederhausbergen, France

**Keywords:** Social behaviour, Animal behaviour

## Abstract

When sharing a space with others, many species including humans evolved a compromise regulating occupancy influenced by social determinants. For example, students in a classroom tend to sit close to their friends, keeping the same spots across days, revealing the social structure in the classroom. This place preference suggests that factors such as social hierarchy and affiliation can shape space utilization, contrasting with random walk models of agents moving at random in any given direction. Here, we asked whether spatial occupancy of macaques within two unisex groups of four individuals, reveals a structured space utilization beyond simple spatial affordance within the finite space. To this end, in two groups of four animals, we analyzed the simultaneously recorded positions of each individual while the group roamed in an enclosure. The data was gathered using automated devices that allow measuring accurate simultaneous positions and calculating precise inter-individual distance, which is impossible in classical ethology, even using GPS devices. Thus, our setup opens new possibilities for modelling approaches, to characterize social interaction dynamics in small enclosures. We found that (1) The identity of each animal could be decoded from its individual pattern of spatial occupancy, revealing that each animal sustained a consistent spatial footprint across multiple days. (2) The average distance between monkeys was a proxy for their social hierarchy, confirming that interpersonal distance is correlated with affiliation and dominance hierarchy. (3) Alternating the social context by removing one of the monkeys revealed that only removing the closest social partner influenced occupancy. (4) Finally, the distribution of distance between pairs of monkeys was bimodal and was modeled using a random walk approach with an additional parameter reflecting the propensity to stay in close proximity, which was again related to dominance hierarchy. These analyses reveal that space utilization is structured as a function of social determinants in macaques and demonstrate the usefulness simple modeling approaches to further study group organization in neuro-ethological settings.

**Supplementary Information:**

The online version contains supplementary material available at 10.1038/s41598-025-16391-w.

## Introduction

How do we manage space socially? A simple measure of the distance between two individuals can reveal their affiliation during social interaction^1^. Indeed, the study of inter-individual distance in humans, defined as proxemics, has grounded the notion that space can be separated into intimate, personal social and public zones^2^ and has given rise to the concept of peripersonal space. Further controlling space in their living environment, in addition to creating shelters, humans often take advantage of physical borders or generate new ones, in order to create private zones which can keep the group together (houses), or divide it (rooms). Although there are cultural and socioeconomic adaptations supporting private divisions within family groups, private space remains a source of conflict within siblings in families and claim of own space is frequent within these groups (“this is my seat, my room”). In some respect, this parallels animals’ territorial behavior regulating space between groups in gregarious species or between individuals in solitary species^3,4^. A behavior is considered territorial when a space is actively defended against intrusion from other animals.

Territorial behavior is manifested in many non-human primates^5^. For example, it is expressed in chimpanzees known to engage into lethal territorial encounters to defend their home ranges^6^. Some other primate species, such as marmosets and lemurs are known to use chemical markings to signal their territories^7^, and also enter a mobbing state when two groups meet^8–10^. In macaques, territorial behavior isn’t clearly manifested and neighbouring groups are known to roam through overlapping home ranges^5,11^. Yet, it has been shown that territorial conflict can emerge depending on seasonal variation in resource availability^12^ or via external manipulation^13,14^. Within the group, how space is shared is actually unknown. It is known that Rhesus and fascicularis macaques show strong agonistic behavior in linked to social dominance^15,16^ and we hypothesize that space occupancy may be a variable affected by this dominance hierarchy. Whether and how space within the group is socially distributed is less known and such information would be of importance given that these animals are the closest experimental model to human used in neuroscience studies^17^. In this study, we took the opportunity to study spatial behaviors in two groups of captive macaques housed together. Thanks to their complex social interactions which rely on finely grained facial expression and grooming to establish and maintain dominance^16,18,19^, rhesus and fascicularis macaques provide an intriguing parallel to human social behavior.

However, little is known about detailed spatial occupancy distribution within a group in macaques because, although tracking of individuals with GPS has improved, positional data within a group isn’t very precise, and social determinants have not often been used as a variable determining space use^20–22^. Therefore, we set out to test whether monkeys exhibited a spontaneous partition of space, and further, how hierarchy and affiliation may be revealed through spatial occupancy. Since space is a limited resource in laboratory animals, we asked whether spatial occupancy can inform us about inter-individual relationships in groups of captive macaque monkeys. We determined whether spatial occupancy of macaques (*Macaca fascicularis* and *M. mulatta*) within two unisex groups, reveals a structured space utilization. We used concurrently collected position data of animals in a cage to identify each monkey’s identity based on their spatial occupancy. We show how it is possible to access the dynamics of individuals in a group.

## Results

### Monkey identity can be decoded from its Spatial occupancy

**Fig. 1:**
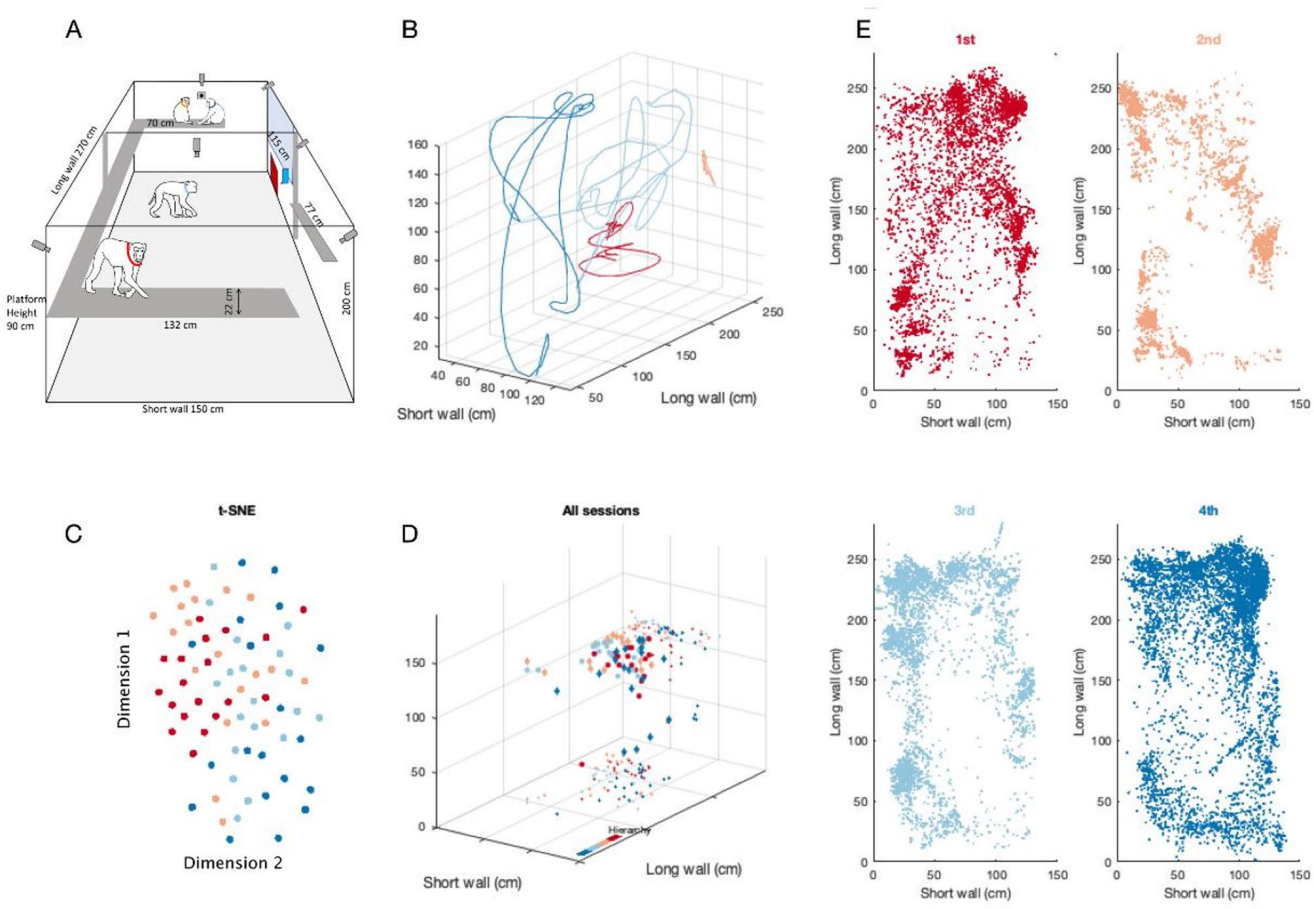
Spontaneous territorial partition of space in a group of four female macaques. (**A**) The home cage and tracking system design, indicating the location of water bottle and fooddispenser. (**B**) The view of the home cage. Example trajectories in 3D of four monkeys from 3 min of recording. The data were smoothed with 10th order Savitzky–Golay smoothing filter. (**C**) To visual the spatial occupancy data across animals and recording sessions the data were projected onto a 2 dimensional manifold using the tSNE method. Each data point represents the data of one individual in a single session. The main observation is that they are not scattered randomly but rather clustered. (**D**) Median positions per session (3 h) per monkey plotted in 3D. The smaller data points depict data projection on the respective wall. Monkeys are coloured according to their hierarchy, from top to bottom, red to blue, respectively. The median positions already appear slightly clustered, suggesting the presence of structured distancing of monkeys in line with their hierarchy. (**E**) Example data from one 3-hour sessions for four monkeys recorded simultaneously. The visual inspection of the patterns suggests that it should be possible to decode monkey identity based on its spatial position.

Via video-based analysis^23^, we computed the simultaneous positions of four female macaques (Group A) co-housed together (see Methods), while they were roaming in an enclosure equipped with video cameras (Fig. 1A and **B**). Data from a second group of four males (Group B) were also collected in the same enclosure independently (**Supp.** Figure 1A). In both groups, the animal’s individual status in the dominance hierarchy (1st to 4th) was estimated during competitive encounters in parallel to the recording sessions (see methods). Averaging movement trajectories for each monkey readily revealed patterns of spatial organization (Fig. 1C-E, **Supp. Figure 1BC**) whereby separation between average positions for each monkey is notable. Visualization of data using low dimensional manifold embedding techniques suggested the same conclusion as the data points representing the 1st and 2nd monkey were clearly grouped near each other (Fig. 1C; **Supp** Fig. 1C) and away from data points representing 3rd and 4th monkeys, which tended to be in the vicinity of each other. These two ways of visual inspection of data suggest an association between space occupancy and monkey identity.

We assessed median positions of monkeys over sessions using Friedman’s ANOVA (Fig. 1D). A Friedman’s test showed that there was a significant difference in the average monkey’s occupancy across sessions in the short horizontal, and vertical wall dimensions, respectively: χ_F_(3, 56) = 18.0, *p* < 0.001; χ^2^_F_(3, 56) = 30.1, *p* < 0.001; however, no significant difference was found along the horizontal wall χ^2^_F_(3, 56) = 5.7, *p* = 0.13. The 3rd monkey occupied a higher vertical position than all the other monkeys (1st: W = 519, Z = 4.3, *p* < 0.001; 2nd: W = 418, Z = 3.2, *p* < 0.01; 4th: W = 502, Z = 3.8, *p* < 0.01). Specifically, the 2nd (113 cm) and 3rd (118 cm) monkeys had the highest median positions, compared to the 1st (98 cm) and 4th (99 cm) monkeys. However, the second monkey still occupied a higher median position than the third monkey (W = 481, z = 3.2, *p* < 0.01). These results suggest that monkey’s occupancy is not random. We hypothesized that dominance hierarchy may be one of the factors that influences monkeys’ pattern of occupancy.

**Fig. 2:**
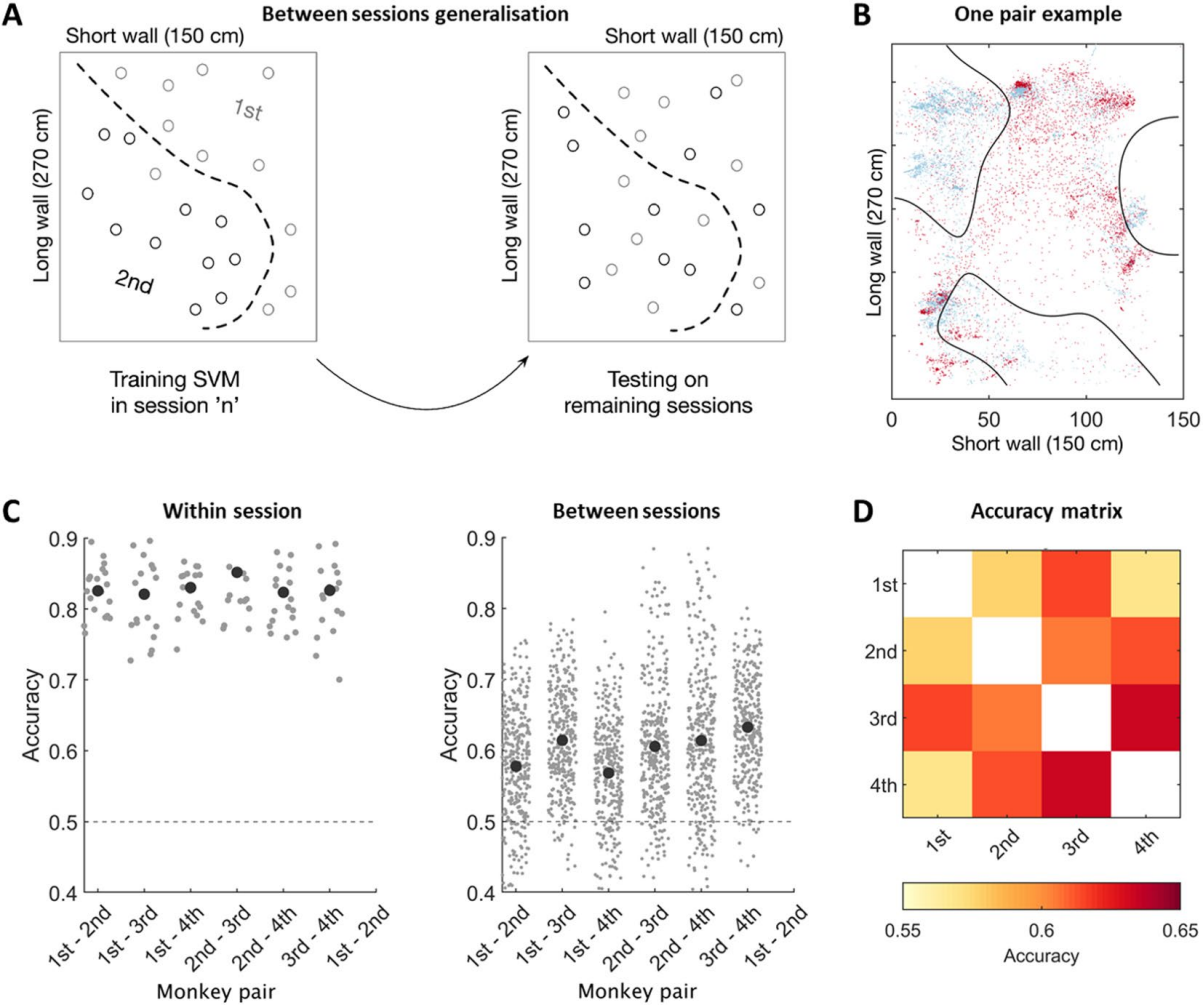
Decoding monkeys’ identity based on spatial occupancy reveals structured pattern of spatial utilization. (**A**) Schematic of the decoding pipeline for monkeys’ identity from their spatial occupancy pattern. We trained the Support Vector Machine classifier (SVM) on the simultaneously recorded monkey positions using 3 dimensions. Using simultaneous positions across time recorded for each monkey pair we decoded monkeys’ identity such that each classifier was trained to distinguish between a pair of monkeys. (**B**) Example of one such trained classifier. The trained classifier was tested in a cross-validated manner (**C**) or on all remaining 18 recording sessions (**D**). (**C**) To decode spatial occupancy within each session and monkey pair, a cross-validation procedure was performed. Decoding accuracy was above the chance level for all monkey pairs (bottom left panel), showing that each animal sustained its spatial footprint within each session. (**D**) The same decoding was performed on data across sessions which showed a similar pattern of results. Left panel: Decoding accuracy was above the chance level for all monkey pairs, demonstrating that each animal sustained its spatial footprint across multiple days. Right panel: Average accuracy scores for each monkey pair are summarized in matrix form in the bottom right panel.

In line with previous analyses, visualization of data from a single example session showed different data patterns across individuals. To quantify the informativeness of this high resolution spatio-temporal patterns, we asked whether the identity of each animal could be decoded from its individual occupancy pattern within recording sessions. To this end, we trained the Support Vector Machine (SVM, Fig. 2A) classifier on the simultaneously recorded monkey positions using 3-dimensional data recorded throughout a session. We performed three kinds of decoding analyses. First, using quadruple sessions we trained and tested classifiers within each of these quadruple sessions with a 10-fold cross validation (Fig. 2BC; Supp. Figure 2A-C). Significant decoding scores signify that occupancy patterns generalize within a single session (Fig. 2C, **Supp.** Figure B, 0.82–0.85 accuracy for all pairs). Second, to assess whether the occupancy patterns generalize across days we trained a decoder on the data from an entire session and tested on all remaining sessions (Fig. 2D, left panel, **Supp.** Figure 2C). Decoding accuracy was above chance level for all monkey pairs (all *p* < 0.001; Fig. 2D, right panel, **Supp.** Figure 2C, **right panel**), showing that each animal sustained its spatial footprint across multiple days. Note that results were qualitatively similar in the second group of macaques (**Supp.** Figure 2).

Next, for group A, we investigated the influence of social context by comparing monkeys’ positions in the absence of a cage-mate on different sessions (Triplet sessions) relative to sessions in which all 4 animals were present (quadruple sessions). This created a different social context for the monkey under consideration by alternating its neighbors. We assessed how the classifier trained on a given pair of monkeys in the quadruple context performs on the same pair of monkeys in the triplet context (**Supp.** Figure 3). Decoding accuracy higher than chance level (all *p* < 0.05) showed that each animal sustained its spatial footprint across multiple days, even when the context changed.

### Quantifying social influence from Spatial occupancy data

**Fig. 3:**
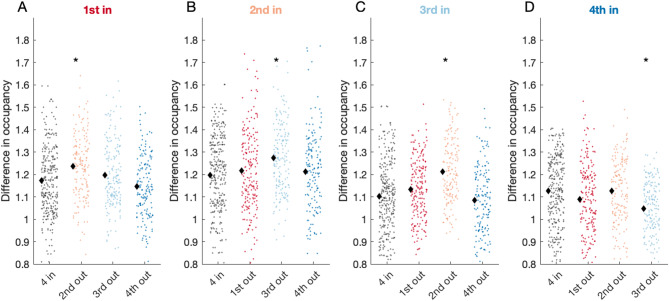
Social context influences monkeys occupancy. Every panel displays the difference in occupancy for sessions with all four monkeys in the home cage (‘4in’), and three kinds of triplets where one of the other three monkeys was removed, therefore creating an effect of social context for the monkey under consideration in one of the four panels. A single data point has been computed by the absolute difference between normalized occupancies from two different sessions originating from the same subject, according to the following formula: mu(|NCn-NCm|), where NC is a 16 by 29 matrix of time spent in a given location normalized by total duration of a recording session. The black diamonds depict the mean for each group, and the asterisk indicates a condition that is different from the ‘4 in’condition.

We aimed to further characterize how the absence of each individual impacts the spatial occupancy of other individuals during the triplet sessions (Fig. 3). One possibility is that only neighboring monkeys in terms of hierarchy would be influenced by the absence of a given monkey. The alternative is that all monkeys would be affected by the absence of a given individual.

Every panel of Fig. 3 represents data of a particular monkey under different experimental conditions. To summarize the change between the quadruple (‘4 in’) session and triplet session in a single number, we computed the L1 normed difference between normalized occupancy maps, which we denote as *D*. Essentially, a single panel in Fig. 3 depicts the amount of change in a given condition with respect to quadruple sessions; except for the first column of each panel of Fig. 3A, which captures to what extent the quadruple sessions vary with respect to other quadruple sessions for a given individual. The latter serves as a baseline level for comparing other conditions. To evaluate the impact of each individual on others’ spatial occupancy, we compared values of *D* between ‘4 in’ sessions and the remaining sessions (colored columns). Significantly different pairs are indicated by an asterisk mark. The 1st monkey in the hierarchy significantly changed its occupancy when the 2nd monkey in the hierarchy was absent (*p* < 0.02; Fig. 3A). However, the 2nd monkey did not significantly change its occupancy when the 1st was absent (*p* > 0.1; Fig. 3B**)**, but instead, was affected by the 3rd monkey’s absence (Fig. 3B, *p* < 0.01). Symmetrically, 3rd monkey was affected by the 2nd monkey’s absence (Fig. 3C, *p* < 0.001). Finally, 4th monkey was only affected by the 3rd monkey’s absence (Fig. 3D, *p* < 0.01, fourth panel).

Overall, social context manipulation allowed us to show that monkeys were modestly, yet significantly influenced only by their closest neighbors’ absence, while other monkeys’ absence had less impact on occupancy structure. Unfortunately, we couldn’t replicate this manipulation on Group B. Yet, these results suggest that depending on which neighbor (higher or lower in hierarchy) exerts influence, measuring behavior in the absence of others, allows us to assess the symmetry and directionality of that influence. However, these results need to be replicated in additional groups.

### Time spent together as a proxy of affiliation

**Fig. 4:**
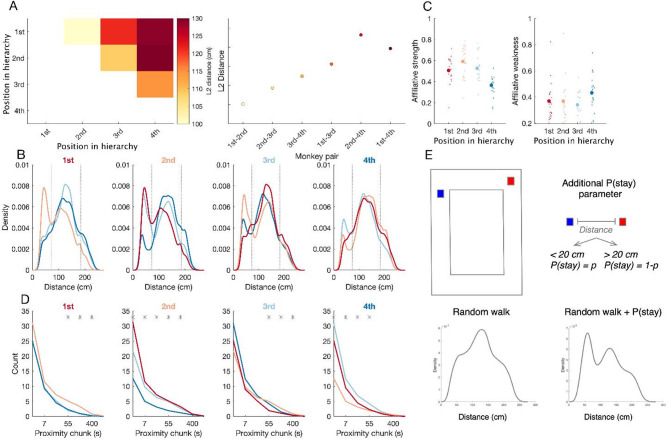
Features of interpersonal distance as a proxy of social structure. (**A**) The matrix represents the average distance for all sessions (*n* = 19) for each pair of monkeys depicted in matrix form. The redness of each cell represents the average distance between a pair of monkeys. Higher values are obtained for 1st and 4th in the hierarchy (top right). The right panel depicts that the physical distance increases as the rank distance between any two monkeys increases. (**B**) We computed Euclidean distance between each pair of monkeys over time. Individual density distribution depicts distribution of distance between a given monkey pair. For example, the first panel displays three density functions which depict the distribution of distance between the 1st monkey and the other three monkeys. The same follows for the three other plots. (**C**) Affiliative strength and affiliative weakness were calculated as the total time each monkey spent with all other monkeys or the total time spent away from other monkeys, respectively. (**D**) Temporal distribution of close proximity chunks. The same color coding follows as in panel A. The top stars indicate the samples where significant differences have been found. (**E**) Schematic and results of random walk model capturing bimodal distributions. The top panel shows schematic of simulation of two agents. The bottom panels display an example simulation involving only random walk and the second one involving random walk with an additional social rule of behavior.

While spatial occupancy of co-housed monkeys revealed individual spatial patterns, we hypothesized that the interpersonal distance between monkeys may reveal the dominance and affiliation structure of the group. First, we computed the overall average distance between monkeys for each session (Fig. 4A, left panel; **Supp.** Figure 4) with a single entry of the matrix showing the average distance between a pair of monkeys. Using t-tests (see methods), we compared the series of instantaneous distances between monkeys for different pairs. All combinations of pairs significantly differed except for a difference between 1st -4th and 2nd -4th (t(18) = 1.9, *p* > 0.1), suggesting that the 4th monkey was equally distant from the first two monkeys in the hierarchy. To illustrate the physical distance between monkeys as a function of the hierarchy, we rearranged the distance matrix in the right panel of Fig. 4A (right panel). In this group, averaged distance increased as the dominance hierarchy distance increased as well. The analyses of the second group of macaques revealed qualitatively similar results (see **Supp. Figure 5 A**).

Next, we examined the distribution of interpersonal distance for each pair of monkeys at a fine resolution (Fig. 4B for group A, and **Supp. Figure 5B** for group B). For each group, there are two peaks in the distribution of distances for at least one pair of interactions per monkey, with the left peak expressing the amount of time spent in close proximity for the given pair. Indeed, significant differences were localized in the left part of the interpersonal distribution (**Supp. Figure 5**,** Supp. Figure 6**). In group A, the monkey at the top of the hierarchy spent a lot of time with the next monkey in the hierarchy. Strikingly, the monkey with the lowest rank spent very little time with other monkeys. In the second group, an overall similar pattern was observed with the presence of a left sided peak for at least one pair of interactions. Contrary to Group A, the peak of close proximity has developed between 1st and 3rd monkeys (**Supp.** Figure 4). In addition, some monkeys in Group B (3rd and 4th monkeys) unexpectedly showed a large right sided peak suggesting avoidance.

The time spent in close proximity with others, can vary from one monkey to another depending on affiliation. To capture differences between animals; we calculated the time spent in close proximity with others (i.e. less than 73 cm away (see Methods for details), defined as affiliative strength (Fig. 4C, left panel). In group A, the affiliative strength of the 4th monkey was smaller than that of the other monkeys (1st : W = 495, Z = 3.62, *p* < 0.001; 2nd : W = 513, Z = 4.15, *p* < 0.001; 3rd : W = 499, Z = 3.74, *p* < 0.001). The 2nd monkey tended to have the strongest affiliative strength, which was larger than the affiliative strength of the 4th monkey (W = 513, Z = 4.15, *p* < 0.001) and nearly that of the 1st monkey (W = 303, Z = 1.96, *p* = 0.051).

Conversely, the analysis of affiliative weakness, that is the time spent away from others (i.e. above > 185 cm, Fig. 4C, right panel), revealed that the 4th monkey spent the least time with the other monkeys (1st : W = 310, Z = 1.75, *p* = 0.079; 2nd : W = 2, Z = 2.07, *p* = 0.038; 3rd : W = 292, Z = 2.27, *p* = 0.023).

Interestingly, these results corroborate the findings of social context manipulation where the 2nd monkey had the most influence over other individuals (Fig. 3). The second observation in line with the findings from social context manipulation is that the proximity peaks occur for the monkeys further apart in the hierarchy. For the monkeys further apart in the hierarchy the close proximity peak is almost negligible. In sum, analysis of affiliative strength adds another dimension to analysis of the dominance hierarchy. A similar pattern regarding affiliative strength and weakness was corroborated by the analysis of the second group of male macaques (**Supp. Figure 5BC**).

The analysis above captures affiliation via the computation of overall time spent together, but does not capture the temporal dynamics of affiliative behavior: when monkeys are close together, how long does this last? To address this question, we analyzed the duration of the bouts of time (chunks) spent in close proximity and analyzed chunk size distribution (Fig. 4D, **Supp. Figure 5D**). It is easy to notice that the chunk size distribution follows the scaling, such that for all pairs of individuals, the number of short interactions is higher than the number of long interactions. Interestingly, different pairs differed in the frequency of those short and long interactions (Fig. 4D, **Supp.** Figure 4C). From this analysis it becomes evident that the peak of close proximity is driven by the large number of long interactions as opposed to a large number of short interactions in both groups of monkeys.

### Accounting for distribution of interpersonal distance

To capture what accounts for the nature of interpersonal distance distribution (Fig. 4A,B), we took a mechanistic approach, by first testing whether the right side of the distribution could be simulated as free roaming behavior. This can be modeled as a two-dimensional random walk process^24–26^; see *Methods: Random walk model simulation*). While the random walk model captured well the right side of the distribution of interpersonal distance (Fig. 4E), it did not capture the peak of close proximity for some monkey pairs. Further, previous analyses suggested that occupancy was not due to purely random walks. Therefore, we included an additional parameter in the previous simulation, expressing the propensity to stay in close proximity once two agents were in close proximity. Precisely, when interpersonal distance was below the threshold, next positions were kept for the next sample according to the parameter’s fitted probability (p(stay), see methods). This simple simulation captured well the distribution of interpersonal distances for pairs showing bimodality in their distribution of pairs, with a high correlation coefficient between real and simulated data (**Supp. Figures 7 & 8**). Although coefficients of correlation were higher, than chance for Group B, this was less the case in Group B, consistent with relatively less time spent close together in this group (**Suppl. Figure 5BD**).

## Discussion

Capturing natural social interactions necessitates, by essence, an understanding of live interactions. In any non-human primate society, these interactions take place in a real physical space, and individual’s positions and distances with respect to each other may reveal the nature of interaction^21^. Here, we took the opportunity to analyze very simple measures in two groups (rhesus and fascicularis): by analyzing the concurrent spatial positions of individuals, we asked how space is jointly shared within the groups. We showed that there is a spontaneous distribution of space between individuals such that an animal’s identity could be decoded from their position (Fig. 2D). While animals could have displayed homogenous occupancy, we showed this was not the case. Animals form inner spatial preferences within the cage enclosure showing that space is socially distributed and further the dynamics of interactions also reveals affiliative patterns within a group. Further, individuals’ spatial preferences remained sufficiently stable to decode their identities across different days (Fig. 2E), and decreased only when decoding was applied on sessions during which the closest monkey in the hierarchy was absent (Fig. 3). Next, we showed that all monkeys displayed social preferences expressed as an increased time spent with some other animals over others (Fig. 4). The close proximity for pairs with a strong affiliation, consists of a few relatively long-time bouts (~ 6 min) during which animals stay close, while most of the time, monkeys spend less than a second in close proximity. Interestingly, the effect of the nearest neighbor (Fig. 3) refers to the individual with the strongest affiliative bond, based on proximity and social interaction patterns (Fig. 4B). The bimodal distribution of interpersonal distances expressing these individual social preferences could be modeled by simple parameters, which is the probability to stay together, added to the simulation of a random walk afforded by the physical structure. While network analysis is usually based on the frequency of interaction, here we show that simple measures of interpersonal distance reveal the complexity of the social network, intertwining dominance hierarchy and affiliation.

### A Spatial footprint for individuals

Macaques aren’t known for a strong territorial behavior, and neighboring groups tolerate overlapping home ranges^12,16^. Further, in field observations, an increase in group size did not impact interpersonal distance within the group^20^, but only increased daily path length, suggesting the latter is a coping strategy relative to heightened competition in larger groups. Here, we reveal that in the absence of food restriction, a simple metric such as an individual’s position within a group, within and across days, supports decoding an individual’s identity. It is also on par with previous work showing that animals have preferences for specific locations within a space^27^. What exactly drives these location preferences is difficult to evaluate in this limited laboratory environment. It has been suggested that high vertical positions might be preempted by higher ranking animals, a hypothesis that we could not really test given the small height of our cages, and the presence of food resources on the floor, which may have inverted that preference. Yet, in this limited cage, an animal’s individual preferences appear akin to a signature footprint, and supports socially distributed space.

One interesting mild but significant effect was that individual spatial occupancy was influenced by the absence of certain other group members. Specifically, the spatial occupancy of a monkey changed the most when its closest social partner was absent. The changes in occupancy were generally larger when comparing triplet sessions to quadruple sessions for all monkeys except for the lowest ranked monkey, for whom these differences were smaller. This suggests that the lowest ranked monkey’s occupancy was more similar within triplet sessions, likely because higher ranked monkeys may have taken over its space. Overall, this suggests some sort of pyramidal control in which behavior is affected by close, rather than distant, interactions.

### Interpersonal distance reveals affiliation

In non-human primates, allogrooming is central to maintaining social bonds^28,29^ and reveals the structure of the social network^30–33^. Here, we show that interpersonal distances reveal affiliation without the cost of scoring grooming as is usually performed. Specifically, the distribution of the interpersonal distance is bimodal for some pairs compared to others, showing that (1) there is a “one arm length” distance that emerges naturally from the distribution, and (2) animals have individual preferences to stay close to some animals over others. The bimodal distribution of interpersonal distances could be modeled by adding a simple parameter to a random walk, such that the probability to remain close was increased when interpersonal distance reached a specific threshold. This shows that the bimodal distribution is not the result of a distribution of movements by physical affordance, but reveals specific affiliations. These specificities were present in both groups, although in the second group, there were also tri-modal distributions, suggesting that some animals also avoided others and tried to stay further away from the others in the enclosure. Thus, the analysis of the distribution of interpersonal distance in a finite space may reveal multiple levels of interactions. The analysis of the durations spent together revealed that for each pair, there were a few bouts of time during which animals stayed together. Specifically, to our surprise, animals rarely spent more than 6 continuous minutes in close proximity. The times during which the data was collected were in the late afternoon while the staff had left and animals were not disturbed. So, our data should reflect monkey’s natural behaviors. While our data is consistent with previous findings showing that macaques gave or received grooming for about 15 min per hour^34,35^, we show that the time spent in close proximity to others, which may include grooming moments, is highly dynamic even in captive monkeys, and is interspersed with other activities.

### Limitations of study

A limitation of this study is the small sample size and its focus on only two macaque groups. Further, when assessing the effect of the absence of individuals on the spatial occupancy of others, we could not always observe a systematic effect, which suggests that both dominance hierarchy and affiliation could play a role in the changes in spatial occupancy. Further experiments should be conducted to tease these factors apart. Performing experimentation on social context in larger groups would also be extremely interesting but difficult. GPS data may not yet provide the level of resolution needed to get access to the fine interpersonal distance that is provided in this study. Yet, although our study is only based on two groups, we found consistencies between the results. Thus, while limited, this study provides valuable insights into socio-spatial behavior, and future research with larger samples and more groups could further enrich our understanding of these dynamics with the proposed tools.

## Conclusion

Overall, the study shows that the dynamics of spatial occupancy reveal the nature of social interaction in macaques consistent with social hierarchy and affiliation. One of the strengths and original contributions of this paper, is that, while territories are usually studied at the group level, here we examined the territory or range at the individual and dyadic levels. As our study was robust across groups and across sessions, our method provides a simple and accessible tool to objectify social relationships within a group revealing interesting interindividual patterns that structure the social network.

## Methods

### Data collection

We recorded monkey positions within a confined space (Fig. 1AB). The size of the experimental space was 150 cm wide, 200 cm high, and 270 cm long and was a subsection of their housing. The perches were placed almost all around the cage at a height of 90 cm. The water bottle was near the ceiling, as indicated in Fig. 1A, in the middle of the long wall. The food dispenser, providing access to crackers, was located in a similar position at floor level.

We used two unisex groups of four monkeys (*Macaca mulatta*,* Macaca fascicularis*). Both groups of animals arrived together at the laboratory and were group housed continuously. Animals were provided from a breeding center in China (Group A, 4 females, 4 years old) or Mauricius Island (Group B, 4 males, 2.5 years old upon arrival to the lab). All experimental procedures were approved by the animal care committee (Department of Veterinary Services, Health & Protection of Animals, permit no. 69 029 0401) and the Biology Department of the University Claude Bernard Lyon 1, in conformity with the European Community standards for the care and use of laboratory animals (European Community Council Directive No. 86–609). Further, our procedures were examined by CELYNE, the local ethics board, from University Lyon. On a daily basis, animals were accustomed to behavioral testing via clicker and positive reinforcement and brought to the laboratory to undergo behavioral operant testing. We started monitoring animals’ positions in the home cage 8 months after their arrival in the laboratory for the Group A and 13 months for the Group B. The tracking of animals in their home cage was performed from 5.00 p.m. to 8.00 p.m. to minimize disturbance to the animals caused by the experimenter’s presence. The animals were housed in a colony room composed of eight other monkeys housed in four adjacent cages of similar size. The animals had continuous access to another adjacent enclosure of approximately the same size, except during the recording sessions, during which they were restricted to the one cage equipped with cameras.

During the recording sessions, monkeys were continuously tracked using 7 digital cameras installed around the cage and oriented so as to minimize blind spots. Each animal’s position was concurrently tracked by at least two cameras with a sub-second resolution (15 Hz) using the collar’s color as an identifier^23^. Before experiments, a calibration of the system was performed with a LED light, and achieved a 0.4 cm resolution. Then, a post processing filter detected each of the animal’s collar with an optimization of the RGB filters. The 3D position reported is the barycenter calculated from the colored pixels detected on the images for each of the collars. We estimate the resolution of the positional precision to be about 5 cm, given the size of the collars is 10 cm diameter. The hardware, software, camera image processing, target triangulation, and automatic calibration are described in full detail in Ballesta et al., 2014.

For the first group of females (*M. mulatta*) we recorded data on 19 separate days. Each recording session lasted 3 h. All the analyses were replicated in a second group of 4 animals (quadruple) for a total of 9 sessions.

To investigate the effects of social context on occupancy we recorded 10 sessions where only three monkeys reside inside the cage (triplet). As there were 4 monkeys that resulted in 4 kinds of triplet sessions.

### Assessment of dominance hierarchy

In order to characterize the dominance relationship of individuals in the group, we performed 8 sessions of a food-grab test through which food was offered to isolated pairs. This was the only way to assess beyond the hierarchy for monkeys # 3 and #4, as when all four monkeys were together, only monkey# 1 and #2 retrieved food. In our tests, the monkey which retrieved the food over all other three was termed #1, while the 2nd retrieved the food over others only in the absence of the highest ranked monkey and so on. The hierarchy of Group B was assessed using the “water bottle test” which scores hierarchy through a similar competition outcome method^36,37^.

### Statistical analyses

We conducted statistical analyses using both parametric and non-parametric tests, depending on the distribution characteristics of the data. For data that did not meet the assumptions of parametric tests, we applied Friedman’s test as a non-parametric alternative and used the Wilcoxon signed-rank test for paired comparisons. The specific tests used are described in the corresponding paragraphs for each analysis. All statistical tests were performed using the Statistics and Machine Learning Toolbox in MATLAB (*v2021a*).

### Decoding analyses

All decoding based analyses were performed using a Support Vector Machine classifier (SVM) on the simultaneously recorded monkey positions using 3 dimensions, using *fitcsvm* MatLab function (*v2021a*). We used a radial basis function kernel. The ‘box constraint’ (aka ‘C parameter’) was set to 1. For within class (quadruples) we used 10-fold cross validation. The data were randomly split along the time axis on a sample-by-sample basis. Specifically, each data point was randomly assigned to either the training set (90% of the data) or the test set (10%), in a non-consecutive manner. For decoding, to assess generalization between quadruple and triplet sessions, we used an entire session for the training and another session for testing. We assessed statistical significance by comparing the results to the same analyses with shuffled labels.

### Computing social influence metrics

For each monkey, session, and condition we computed normalized occupancy maps expressing the proportion of time spent in a given part of the area. To summarize between sessionchanges we computed a single number index as the L1 normed difference between normalized occupancy maps, depictedas a single data point in Fig. 2A. We computed the L1 normed difference between all combinations of quadruple and triplet sessions (Fig. 2A, colored column in each panel). Such index (*D*) shows the amount of change between the quadruple and triplet context for a particular monkey, quantifying the amount of change for a given monkey under a particular social context. We also computed the L1 normed difference between all combinations of quadruple sessions (Fig. 2A, black column in each panel), which we treat as a reference level for changes observed in triplet sessions.

As the number of triplet sessions differed between monkeys, and thus also between context conditions, we accounted for that fact using a variant of permutation testing. For each permutation sample, we randomly chose 100 quadruple and 100 triplet values of *D.* We repeated that procedure 200 times, which resulted in a distribution of *t* and *p* values. We based our inference on the average value of *p*.

### Assessment of affiliative strength and affiliative weakness

The thresholds were determined from the average distribution of interpersonal distances, using the first local minimum following the close-proximity peak and the first local minimum after the peak on the right side of the distribution. As a result, affiliative weakness was measured as the amount of time spent at distances greater than 185 cm from others and affiliative strength was measured as the amount of time spent at distances smaller than 73 cm. We first computed Euclidean distance for each sample for a given pair of monkeys, resulting in one time series for a given pair of monkeys (Fig. 4A). As such that analysis neglects the absolute spatial positioning of individuals. To compute the affiliative strength for each session we calculated the cumulative sum of distance for each pair of monkeys. We summed up this metric for all three pairs for each individual. We calculated the cumulative sum over distance ranging from 0 to 80 cm, which approximately captured the peak of close proximity. We then compared this metric using sessions as samples.

### Assessment of close proximity chunks

To identify the chunks of close proximity, where two individuals were separated by less than 50 cm we counted the consecutive number of samples that fell into that criterion. In case of an individual sample, it was considered as a one sample chunk. To facilitate the visualization of results, we converted the time scale to logarithmic scale to calculate histograms. Finally, different histograms (Fig. 4D) were compared within different intervals using Kolmogorov-Smirnov test.

### Random walk model simulation

To simulate monkeys’ movement with random walks we set up a 240 by 140 steps grid lattice. Based on the empirical data we knew that monkeys spend most of their time on the boundaries of the colony room, thanks to the usage of perches. Thus, we constrained the lattice such that agents can move in the vicinity of 40 units, perpendicular to the wall. At each step the agents would move in a random direction. To account for variable speed of movement the size of the step was randomized from uniform distribution from 1 to 40 in steps of 1. We simulated 10^4^ random walk samples. We then calculated Euclidean distance between a pair of agents.

In the second simulation we aimed at modeling the peak of close proximity as random walk model did not capture bimodal distributions. Therefore, we enhanced the random walk model with the addition of a simple rule. On every sample that two agents were in close proximity (< 10) the new parameter expressed the probability of staying in close proximity on the next sample (P(*StayTogether*)). We ran separate simulations with the values of P(*StayTogether*) ranging from 0.1 to 0.9 in steps of 0.05.

## Supplementary Information

Below is the link to the electronic supplementary material.

Supplementary Material 1

## Data Availability

Data and codes are available in the following repository https://osf.io/zhg28/.
