# Supplementary material for: Spatio-temporal dynamics of ingroup interactions in macaques
Source: Sci Rep. 2025 Aug 22;15:30913. doi: 10.1038/s41598-025-16391-w (PMC12373813; doi:10.1038/s41598-025-16391-w)
Supplement: Supplementary file 1 — Supplementary Material 1 [file 41598_2025_16391_MOESM1_ESM.docx]

**Supplementary Figures**


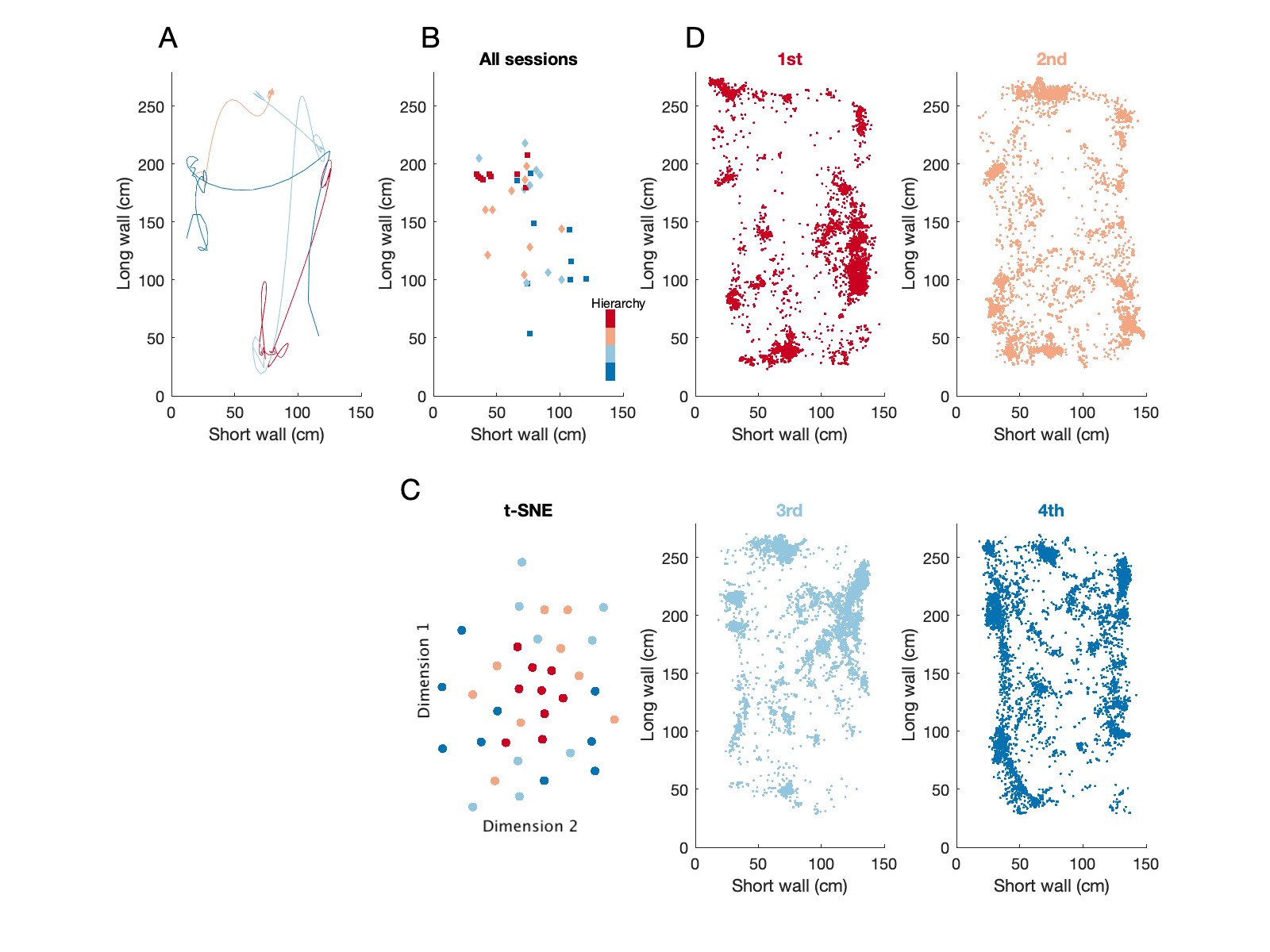


**Supplementary Figure 1.** Spontaneous territorial partition of space in Group B.

(A) The floor view of the home cage. Example trajectories of four monkeys from 3 minutes of recording. The data were smoothed with 10^th^ order Savitzky-Golay smoothing filter.

(B) Median positions per session per monkey plotted in a floor view. Monkeys are coloured according to their hierarchy, from top to bottom, red to blue, respectively. Clustering of median positions suggested that we should have observed structured distancing of monkeys in line with their hierarchy. As for the group of females, we assessed averaged distances between pairs of monkeys over sessions using ANOVA comparisons across each of three dimensions. A Friedman’s test showed that there was significant difference in average monkey’s occupancy across sessions in short wall dimensions: χ^2^(3, 24) = 11.9, *p* = 0.008; but not for long and vertical dimensions, respectively: χ^2^(3, 24) = 6.2, *p* = 0.102; χ^2^(3, 24) = 5.4, *p* = 0.144.

(C) tSNE decomposition Of individuals in Group B. Single data point represents data of one individual in a single session. The main observation is that they are not scattered randomly but rather clustered.

(D) Example data from one session for four monkeys recorded simultaneously. The visual inspection of patterns suggest that it should be possible to decode monkey identity based on its spatial position.


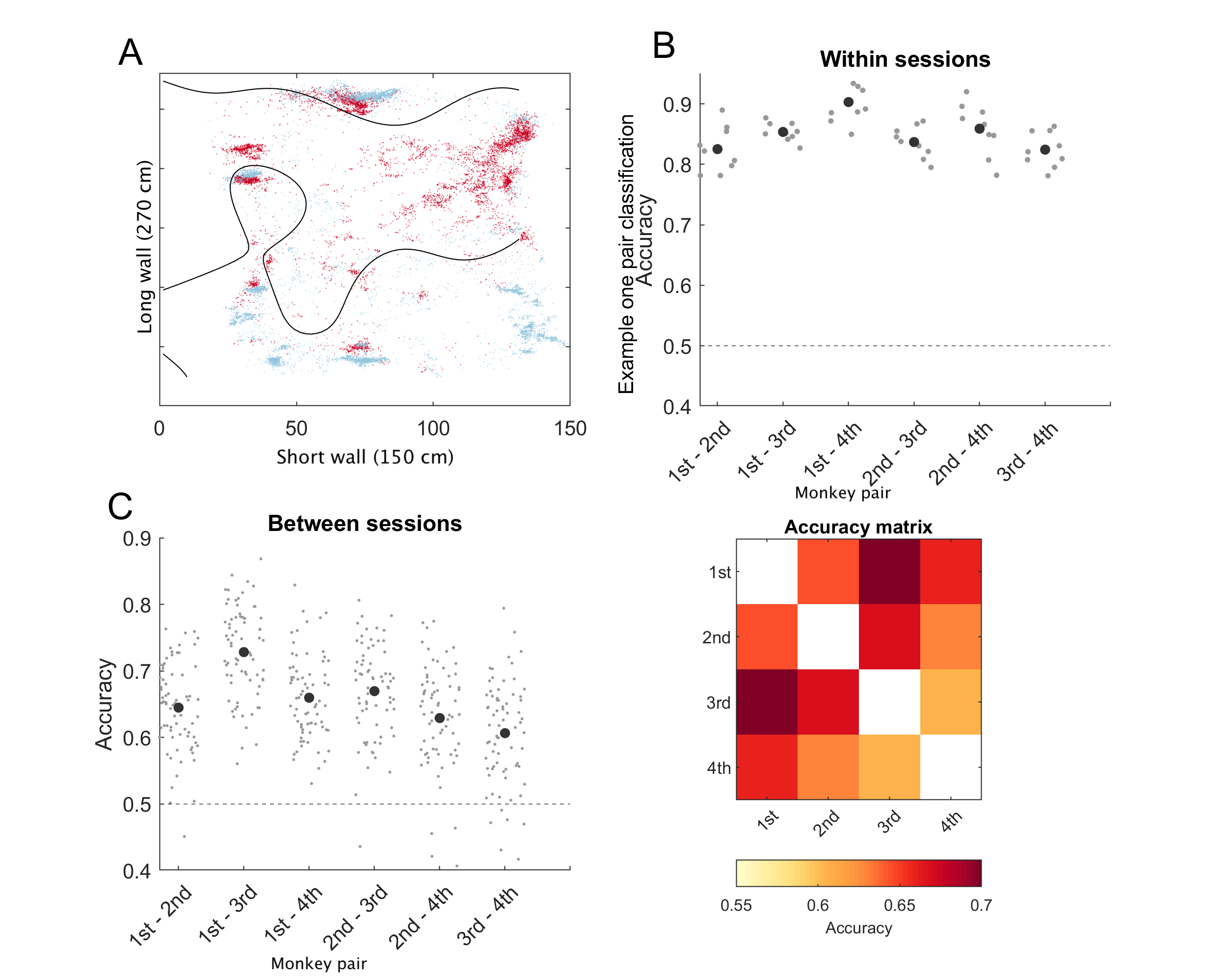


**Supplementary Figure 2.** Decoding monkeys’ identity based on spatial occupancy in Group B.

(A) Example of one such trained classifier. The trained classifier was tested in a cross validated manner or on all remaining 18 recording sessions.

(B) To decode spatial occupancy within each session and monkey pair cross-validation procedure was performed. Decoding accuracy was above the chance level for all monkey pairs (bottom left panel), showing that each animal sustained its spatial footprint within each session.

(C) The same decoding was performed on data across sessions which showed a similar pattern of results. Decoding accuracy was above the chance level for all monkey pairs, demonstrating that each animal sustained its spatial footprint across multiple days. Average accuracy scores for each monkey pair are summarized in matrix form in the bottom right panel.


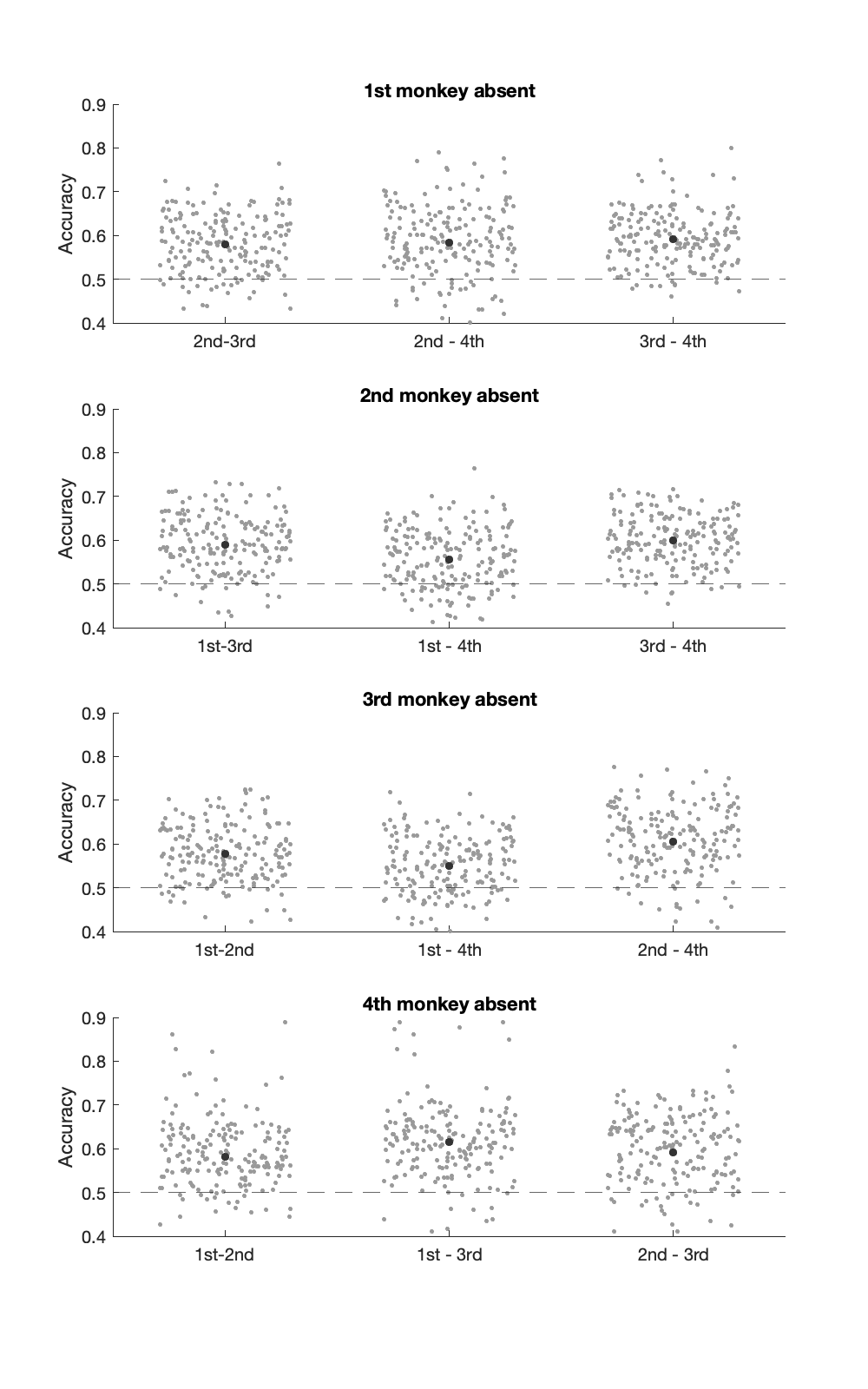


**Supplementary Figure 3.** Decoding spatial occupancy between quadruple and triplet sessions. Classifiers were trained on quadruple sessions and tested on triplet sessions. Decoding accuracy was above the chance level for all monkey pairs, showing that each animal sustained its spatial footprint despite removal of one of the monkeys.


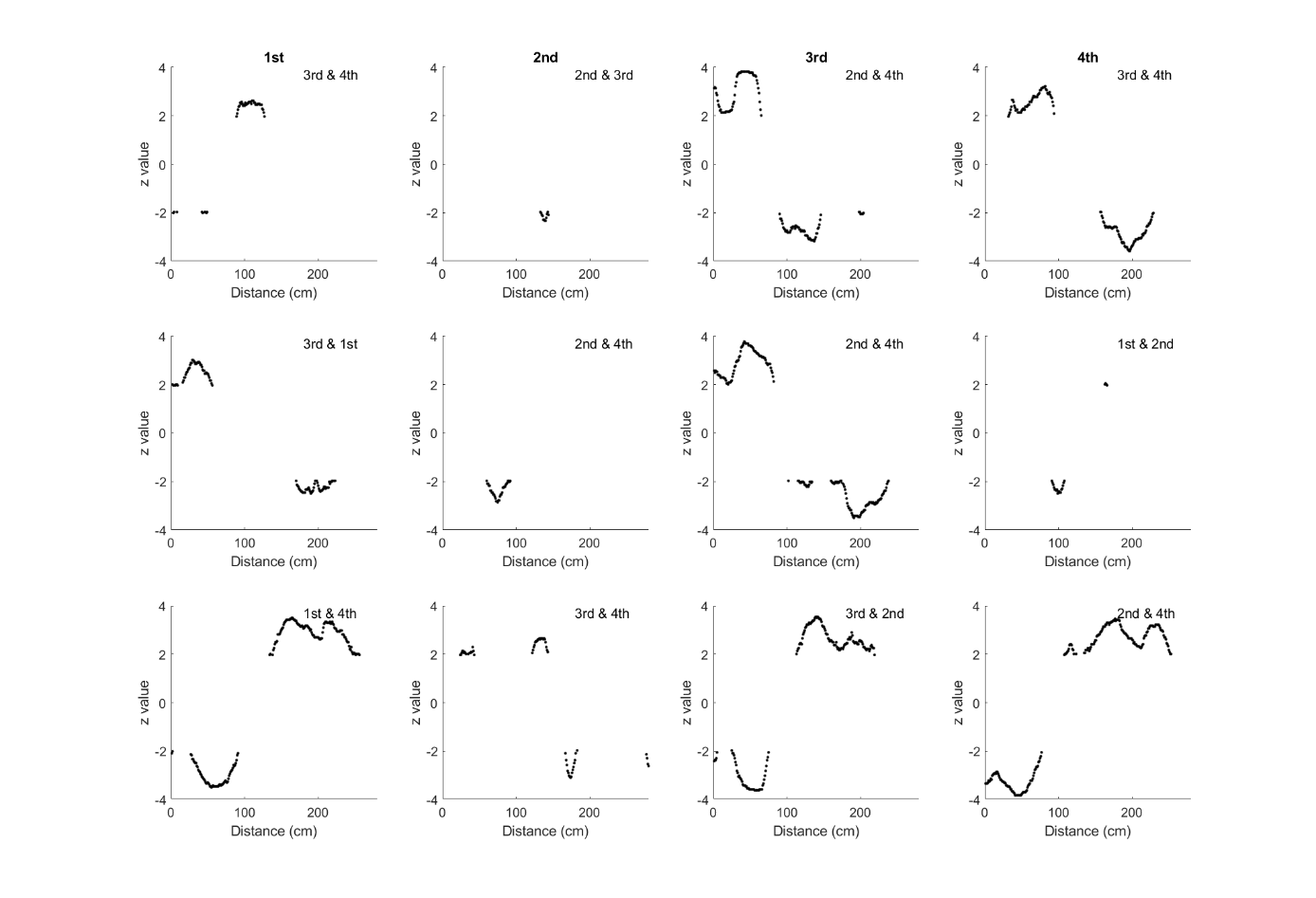


**Supplementary Figure 4.** Comparison of density distributions plotted in Figure 4B for Group A. The columns of the grid plot correspond to one monkey. The rows correspond to a second monkey from the pair. Time axis corresponds to the time axis in Figure 4B.


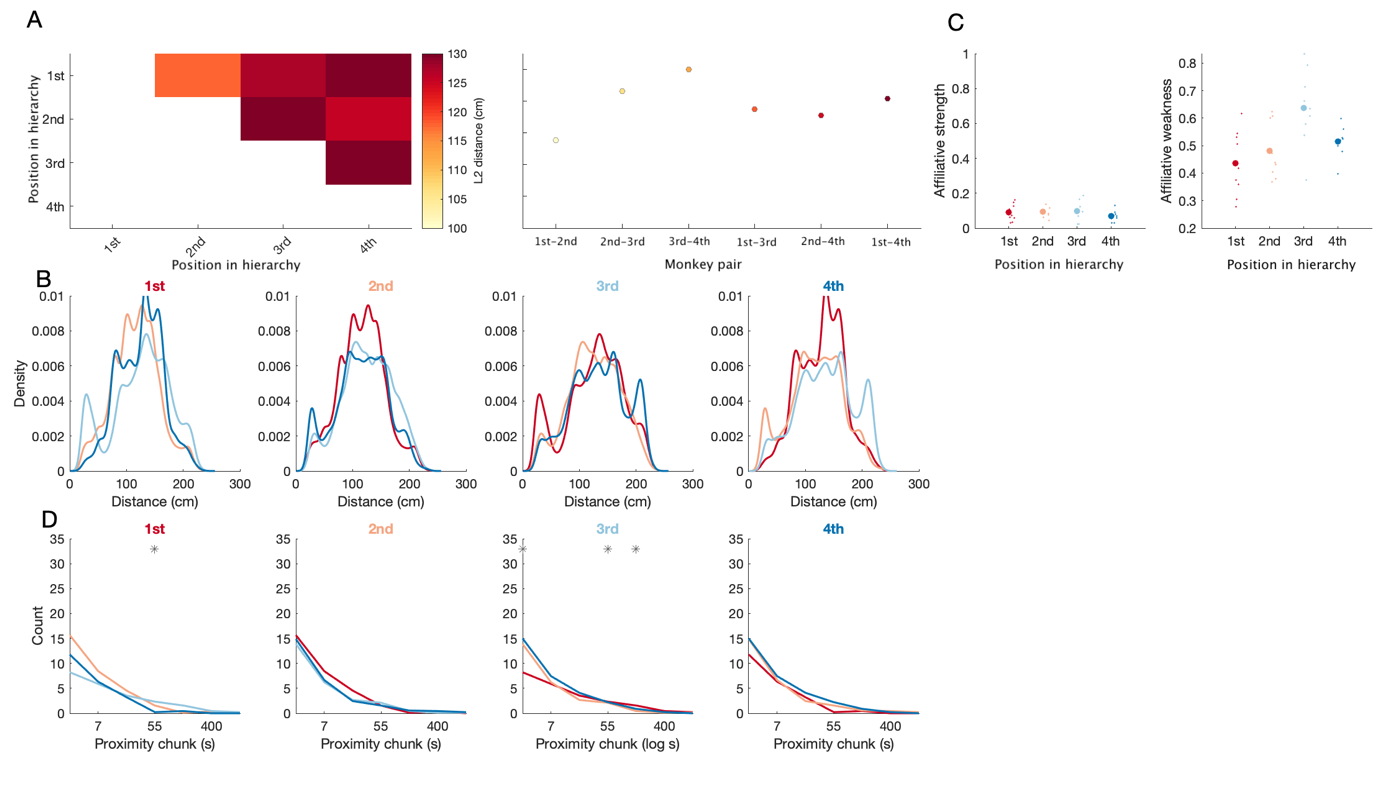


**Supplementary Figure 5.** Features of interpersonal distance as a proxy of social structure in Group B.

(A) The matrix represents the average distance for all sessions (n=9) for each pair of monkeys depicted in the matrix form. The redness of each cell represents the average distance between a pair of monkeys. Higher values are obtained for 1^st^ and 4^th^ in the hierarchy (top right).

(B) We computed the Euclidean distance between each pair of monkeys over time. Individual density distribution depicts distribution of distance between given monkey pairs. For example, the first panel displays three density functions which depict the distribution of distance between 1st monkey and the other three monkeys. The same follows for the three other plots.

(C) Affiliative strength and affiliative weakness calculated as total time each monkey spent with all other monkeys or total time spent away from other monkeys, respectively. Monkeys did not differ in the values of affiliative strength (all p > 0.05). Affiliative weakness was highest for the 3rd monkey as compared to all other monkeys (1st : W = 55, Z =2.65, p = 0.008; 2nd: W = 61, Z =2.11, p = 0.034;4th: W = 114, Z = 2.47, p = 0.013).

(D) Temporal distribution of close proximity chunks. The same color coding follows as in panel A. The top stars indicate the samples where significant differences have been obtained.

**
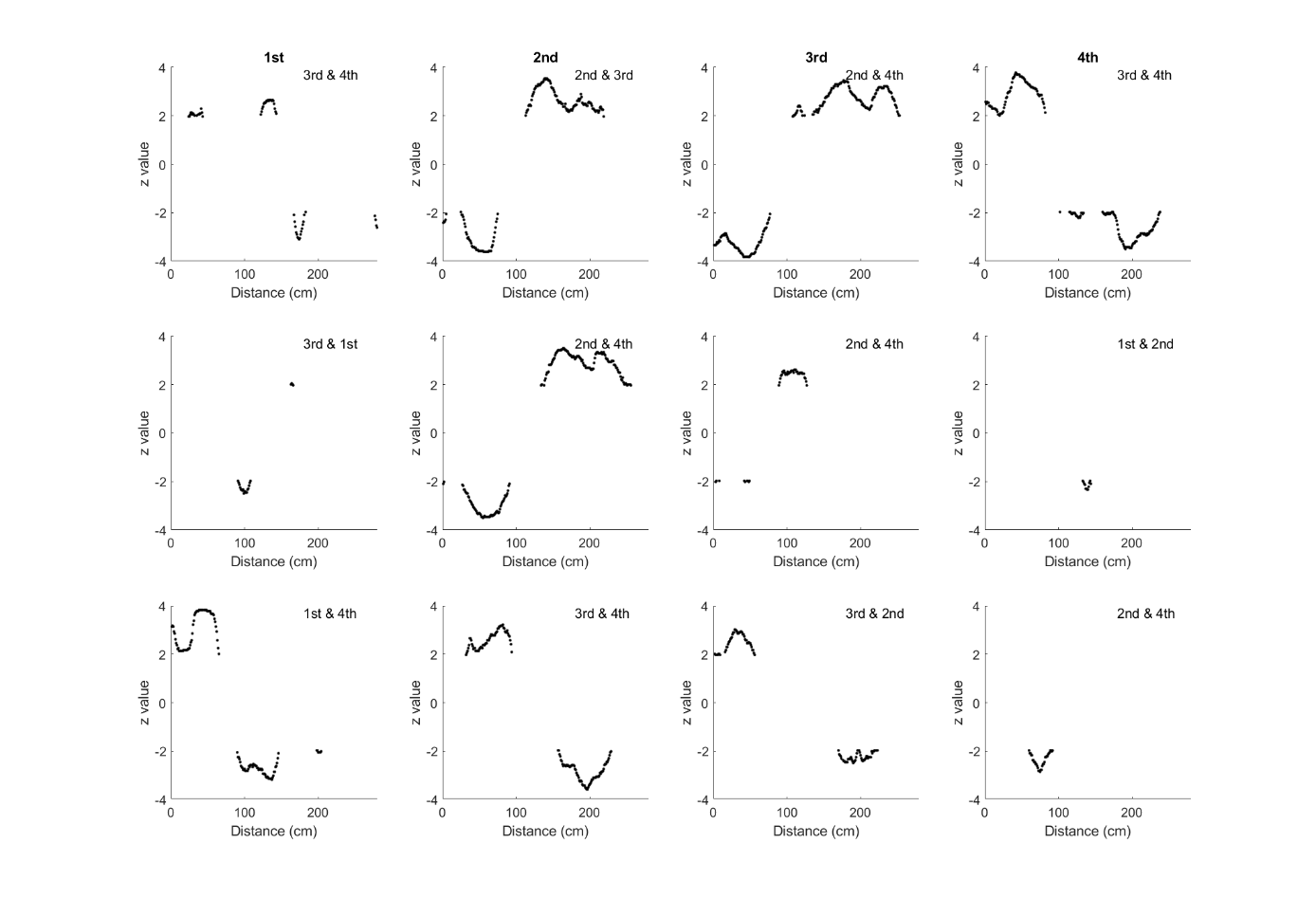
**

**Supplementary Figure 6.** Comparison of density distributions plotted in Supplementary Figure 5B for Group B. The columns of the grid plot correspond to one monkey. The rows correspond to a second monkey from the pair. Time axis corresponds to the time axis in Supplementary Figure 5B.

*
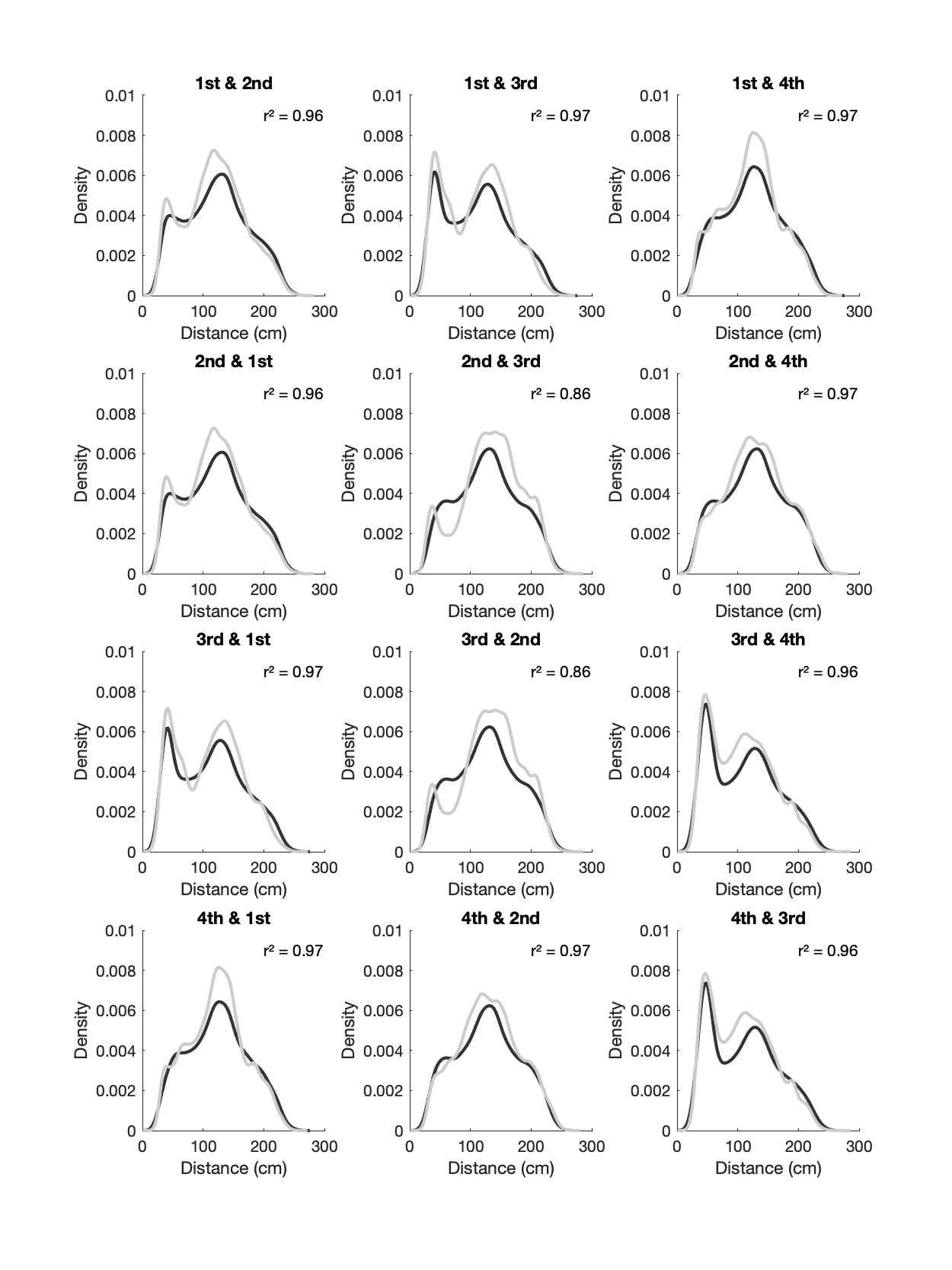
*

**Supplementary Figure 7.** Model fits for Group A. Each distance distribution for a given monkey pair was fitted with simulated distribution with best fitting parameters. Empirical distributions are plotted in grey, whereas the simulated once in black for each pair of monkeys.


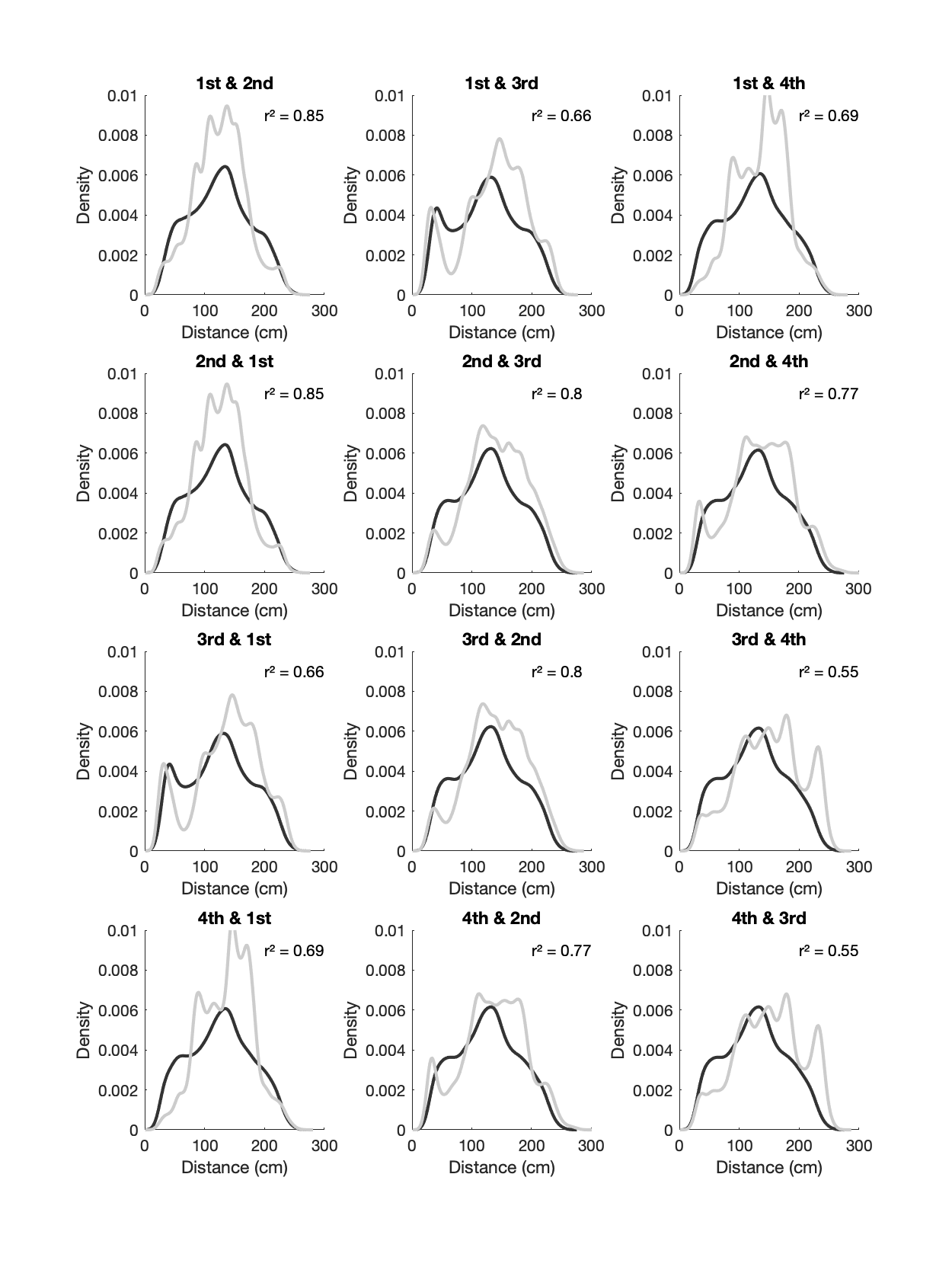


**Supplementary Figure 8.** Model fits in the group B. All details remained the same as in the group A.
